# Supplementary material for: Complexome profiling of the Chlamydomonas psb28 mutant reveals TEF5 as an early PSII assembly factor
Source: Plant Cell. 2025 Mar 17;37(6):koaf055. doi: 10.1093/plcell/koaf055 (PMC12164590; doi:10.1093/plcell/koaf055)
Supplement: koaf055_Supplementary_Data [file koaf055_supplementary_data.zip › psb28_interactive.zip!/plots/intensity/qualityPlots/peptides/IPY1_Cre10.g424100.t1.2.html]

### Cre10.g424100.t1.2

Trivial name: IPY1  
SeqCoverage [median]: 13.20  
Mass: 31.13 kDa  
  
 

| Sequence | MissedCleavage | Mass [Da] | wtIntensity | muIntensity | Score |
| --- | --- | --- | --- | --- | --- |
| ANSEELSLI | 0 | 974.49 | 586.05 | 0.00 | 71.34 |
| MEVATDEPR | 0 | 1046.47 | 906.74 | 618.50 | 141.52 |
| TPIKQDVKK | 2 | 1055.63 | 0.00 | 470.86 | 68.89 |
| VIAISADDPK | 0 | 1027.56 | 402.75 | 3017.32 | 81.55 |
|  |  |  | 1895.5 | 4106.7 |  |
